# Optimal Cut-Offs for Digital Addiction Scales as Preliminary Screening Proxies for Psychological Distress Symptoms Among Vietnamese Medical Students: A Single-Centre ROC Curve Analysis

**DOI:** 10.3390/ijerph23080950

**Published:** 2026-07-23

**Authors:** Linh My Duong, Nghia Quang Bui, Lam Phuc Duong, Ry Thi Khao Duong

**Affiliations:** 1 Faculty of Medicine, Can Tho University of Medicine and Pharmacy, Can Tho 94000, Vietnam; bqnghia@ctump.edu.vn (N.Q.B.); dtkry@ctump.edu.vn (R.T.K.D.); 2 Faculty of Public Health, Can Tho University of Medicine and Pharmacy, Can Tho 94000, Vietnam; dplam@ctump.edu.vn

**Keywords:** depression, anxiety, stress, problematic smartphone use, social media use, gaming-related problems, medical students, ROC curve

## Abstract

**Highlights:**

**Public health relevance—How does this work relate to a public health issue?**

- Psychological distress, including stress, depression, and anxiety, is common among medical students, who may be particularly vulnerable because of academic pressure, clinical training demands, and professional expectations.
- This study examines problematic smartphone use, social media use, and gaming-related problems as potentially modifiable behavioral correlates of psychological distress in young adults.

**Public health significance—Why is this work of significance to public health?**

- The study derives provisional ROC-based cut-offs for digital-use scales and evaluates how well these scores distinguish students with normal versus non-normal DASS-21 symptom classifications.
- The findings indicate that digital-behavior scores may have limited adjunctive value within scalable university mental health assessment pathways.

**Public health implications—What are the key implications or messages for practitioners, policy makers and/or researchers in public health?**

- Universities should not use these thresholds as stand-alone screening or diagnostic criteria; a positive result may instead prompt assessment with a validated mental health instrument or clinical evaluation.
- Longitudinal, multicenter, and externally validated studies are needed to confirm the thresholds, clarify temporal relationships, and inform healthier digital-engagement policies.

**Abstract:**

Digital technologies are central to student life, but evidence is limited on whether digital-use scales can provide useful thresholds for identifying psychological distress among medical students. This single-center cross-sectional study analyzed complete data from 600 undergraduates at a medical university in southern Vietnam. Stress, depression, and anxiety symptoms were classified using the DASS-21, while problematic smartphone use, social media use, and gaming-related problems were assessed using the SABAS, BSMAS, and a six-item GAS adaptation, respectively. Receiver operating characteristic analyses and the Youden index were used to estimate discrimination and candidate cut-offs. Non-normal DASS-21 scores were observed for stress (30.4%), depression (37.8%), and anxiety (39.0%), predominantly within the mild range. For stress, the AUCs and cut-offs were 0.75 (95% CI 0.70–0.80; ≥23.5) for SABAS, 0.67 (0.62–0.71; ≥17.5) for BSMAS, and 0.64 (0.60–0.70; ≥15.5) for GAS. For depression, the corresponding values were 0.62 (0.57–0.66; ≥21.5), 0.68 (0.63–0.73; ≥18.5), and 0.60 (0.55–0.65; ≥14.5). For anxiety, they were 0.60 (0.55–0.64; ≥20.5), 0.62 (0.57–0.67; ≥17.5) and 0.55 (0.51–0.60; ≥15.5). Sensitivity was low across thresholds (35.0–59.0%). These candidate cut-offs showed modest discrimination and should be considered only as preliminary behavioral flags prompting validated mental health assessment, not as stand-alone screening or diagnostic criteria.

## 1. Introduction

Digital technologies, including smartphones, social media platforms, and online games, are embedded in university life. Problematic engagement with these technologies has been associated with stress, depression, anxiety, sleep disruption, and social difficulties [1,2,3,4]. Among young adults, greater social media use has also been associated with perceived social isolation and depressive symptoms [5,6]. Medical students may be especially vulnerable to psychological distress because of demanding curricula, competitive learning environments, and exposure to clinical stressors [7,8,9,10]. Identifying accessible behavioral indicators that co-vary with distress could therefore support timely referral within university health services, provided that such indicators are not mistaken for diagnostic measures.

Problematic smartphone use, problematic social media use, and gaming-related problems are related but conceptually distinct behaviors. Smartphone measures capture broad device-level dysregulation, whereas social media and gaming measures focus on specific online activities [11,12,13,14,15]. Gaming disorder is recognized in the ICD-11 when a persistent pattern of impaired control and functional impairment is present [16]; problematic smartphone and social media use are not equivalent diagnostic entities. Compensatory Internet Use Theory further proposes that some individuals may engage excessively with digital media to cope with negative affect or stressful circumstances [17].

Most previous studies have examined associations between digital overuse and mental health outcomes rather than the ability of digital-use scores to distinguish between symptom categories. The DASS-21 is a widely used self-report measure of depression, anxiety, and stress symptoms, with Vietnamese psychometric evidence available in community and adolescent populations [18,19,20]. ROC analysis can quantify how well a continuous score separates two prespecified groups and can identify a threshold that balances sensitivity and specificity [21,22,23]. However, when both the index measure and the reference classification are self-reported, the resulting AUC reflects empirical discrimination and concordance rather than clinical diagnostic accuracy.

Guided by Compensatory Internet Use Theory [17], this study evaluated the discriminative performance and candidate cut-off values of the Smartphone Application-Based Addiction Scale (SABAS), Bergen Social Media Addiction Scale (BSMAS), and a six-item adaptation of the Game Addiction Scale (GAS) for distinguishing normal from non-normal DASS-21 classifications among medical students. Because DASS-21 categories were used as a symptom-based reference rather than a clinical diagnosis, the digital-use scales were evaluated as preliminary behavioral proxies for psychological distress, not as diagnostic tests.

## 2. Methods

### 2.1. Study Design and Participants

This single-center cross-sectional study was conducted among full-time undergraduate medical students at a medical university in southern Vietnam. The official student roster supplied by the Office of Undergraduate Education served as the sampling frame. Using a computer-generated random sequence, 1000 eligible students were selected and invited through their university email accounts to complete a secure, anonymous online questionnaire.

Eligible students were enrolled full-time, provided electronic informed consent, and completed all questionnaire modules. The invitation stated that participation was voluntary, confidential, and unrelated to academic assessment. Independent research assistants, who had no teaching or grading role for the invited students, distributed invitations, monitored response rates, and sent standardized email reminders.

Of the 1000 invited students, 960 accessed and submitted the questionnaire. A complete-case approach was used for the primary analyses. In total, 360 submissions were excluded because one or more primary variables or multi-item scale responses were missing or because prespecified response-quality criteria were not met. The final analytical sample, therefore, comprised 600 students. This transition from the randomly selected invitation sample to the complete-case analytical sample introduced potential self-selection and attrition bias.

The prevalence-based sample-size calculation used:

$$n=\frac{Z_{1-\frac{\propto }{2\;}}^{2}\;p\;(1-p)}{d^{2}}$$

With *Z* = 1.96 for a 95% confidence level, an expected prevalence of 48.4% based on a 2022 study of depressive symptoms among medical students at Tay Nguyen University [24], and an absolute precision of 5%; the minimum required sample size was 384 participants. The final analytical sample of 600 exceeded this minimum.

No separate a priori sample-size calculation was undertaken specifically for the ROC analyses. The adequacy of the final sample for these exploratory analyses was therefore assessed from the precision of the observed AUC estimates. For stress, depression, and anxiety, the non-normal DASS-21 groups comprised 182, 227, and 234 participants, respectively, whereas the corresponding normal groups comprised 418, 373, and 366 participants. Across the nine ROC analyses, the half-widths of the 95% CIs around the AUC estimates were approximately 0.04–0.05, indicating reasonably precise within-sample estimates of discriminative performance. These precision estimates do not establish external validity or clinical diagnostic accuracy.

### 2.2. Instruments

Data were collected using a self-administered questionnaire comprising demographic items and Vietnamese-language versions of the study instruments. The validation status and adaptation procedures differed across instruments and are described below.

Problematic smartphone use was assessed using the Smartphone Application-Based Addiction Scale (SABAS), a six-item measure scored on a six-point Likert scale [25]. Vietnamese studies have reported psychometric evidence for the related Smartphone Addiction Scale–Short Version (SAS-SV) [26,27], but these studies do not constitute direct validation of the SABAS. Accordingly, the SABAS results in the present study were interpreted cautiously, and internal consistency was evaluated in the analytical sample.

Problematic social media use was assessed using the Bergen Social Media Addiction Scale (BSMAS), a six-item measure reflecting salience, tolerance, mood modification, withdrawal, conflict, and relapse [28,29]. Items are scored on a five-point Likert scale. The BSMAS has been used in Vietnamese young people, with evidence supporting its internal consistency and construct measurement in this context [30].

Gaming-related problems were assessed using a six-item adaptation based on the Game Addiction Scale (GAS) [31]. Because a formally validated Vietnamese version of this six-item adaptation was not available, the items underwent forward translation, expert review, and back-translation before administration. Internal consistency was assessed in the current sample. The resulting scores should therefore be interpreted as study-specific rather than as a formally validated Vietnamese GAS short form.

Psychological distress symptoms were assessed using the 21-item Depression Anxiety Stress Scales (DASS-21), which contains three seven-item subscales referring to symptoms experienced during the previous week [18,19]. Each item is scored from 0 to 3; subscale totals were multiplied by two and classified as normal, mild, moderate, severe or extremely severe according to the standard scoring framework. Vietnamese studies have supported the reliability and validity of the DASS-21 in community and adolescent populations [20,32].

Internal consistency was acceptable to excellent in the present sample. Cronbach’s alpha was 0.821 for SABAS, 0.849 for BSMAS, and 0.900 for the six-item GAS adaptation. For the DASS-21, alpha coefficients were 0.832 for depression, 0.823 for stress, and 0.838 for anxiety.

### 2.3. Statistical Analysis

Descriptive analyses were performed in IBM SPSS Statistics version 20.0 (IBM Corp., Armonk, NY, USA). Youden’s index was calculated in Microsoft Excel 2013 (Microsoft Corp., Redmond, WA, USA). All analyses used complete cases.

ROC analyses evaluated the ability of each continuous digital-use score to distinguish students classified as normal from those classified as non-normal (mild to extremely severe) on each DASS-21 subscale. AUCs with 95% confidence intervals (CIs) summarized discrimination. AUCs were interpreted as excellent (0.90–1.00), good (0.80–<0.90), fair (0.70–<0.80), poor (0.60–<0.70), or failing/near chance (0.50–<0.60) [21,22]. Candidate thresholds maximized Youden’s index (J = sensitivity + specificity − 1). Sensitivity, specificity, positive predictive value (PPV), and negative predictive value (NPV) were calculated at each threshold; 95% CIs for sensitivity and specificity were estimated using the Wilson method. Statistical significance was defined as *p* < 0.05. Because the nine ROC analyses were exploratory, no adjustment for multiple comparisons was applied; interpretation therefore emphasized AUC magnitude and 95% CIs rather than nominal *p*-values alone.

### 2.4. Ethical Approval

The study was conducted in accordance with the Declaration of Helsinki and was approved by the Biomedical Research Committee of Can Tho University of Medicine and Pharmacy, Vietnam (approval no. 24.025.GV/PCT-HĐĐĐ; 20 May 2024). Electronic informed consent was required before participants could access the questionnaire.

## 3. Results

### 3.1. Sociodemographic Characteristics of the Sample

The analytical sample comprised 600 students (Table 1). The mean age was 22.9 ± 3.7 years (range 20–48 years). The wide age range reflected the inclusion of both conventional direct-entry students and non-traditional work-study or continuing-education students who had returned to medical training after clinical practice. Women represented 52.7% of the sample. Third-year students formed the largest academic-year group (41.2%), and 58.7% reported adequate household income. Economic status was collected for descriptive purposes and was not included as a covariate in the ROC analyses.

### 3.2. Prevalence of Stress, Depression, and Anxiety Symptoms

Table 2 presents the distribution of DASS-21 symptom categories. Most students were classified as normal for stress (69.6%), depression (62.2%) and anxiety (61.0%). Non-normal classifications were therefore observed in 30.4%, 37.8% and 39.0%, respectively. Mild symptoms accounted for most non-normal classifications: 17.7% for stress, 24.0% for depression and 26.5% for anxiety. Moderate symptoms occurred in approximately one-tenth of the sample for each domain, while severe and extremely-severe categories were uncommon.

### 3.3. Receiver Operating Characteristic Curve Analysis

**Figure 1:**
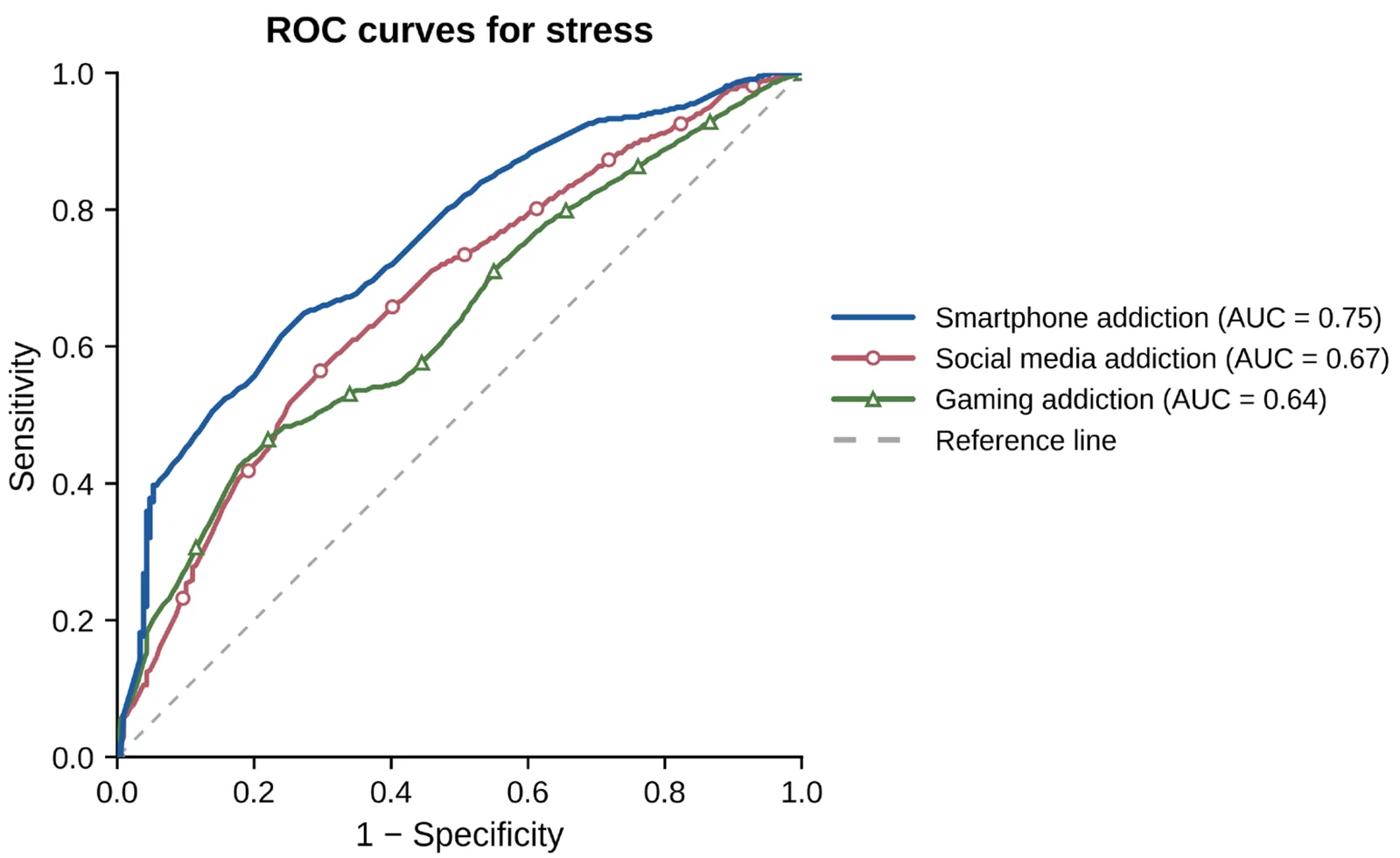
ROC curves of digital technology addiction for stress.

For stress, SABAS showed fair discrimination (AUC = 0.75, 95% CI 0.70–0.80; *p* < 0.001). BSMAS (AUC = 0.67, 95% CI 0.62–0.71; *p* < 0.001) and the six-item GAS adaptation (AUC = 0.64, 95% CI 0.60–0.70; *p* < 0.001) showed poor discrimination (Figure 1).

**Figure 2:**
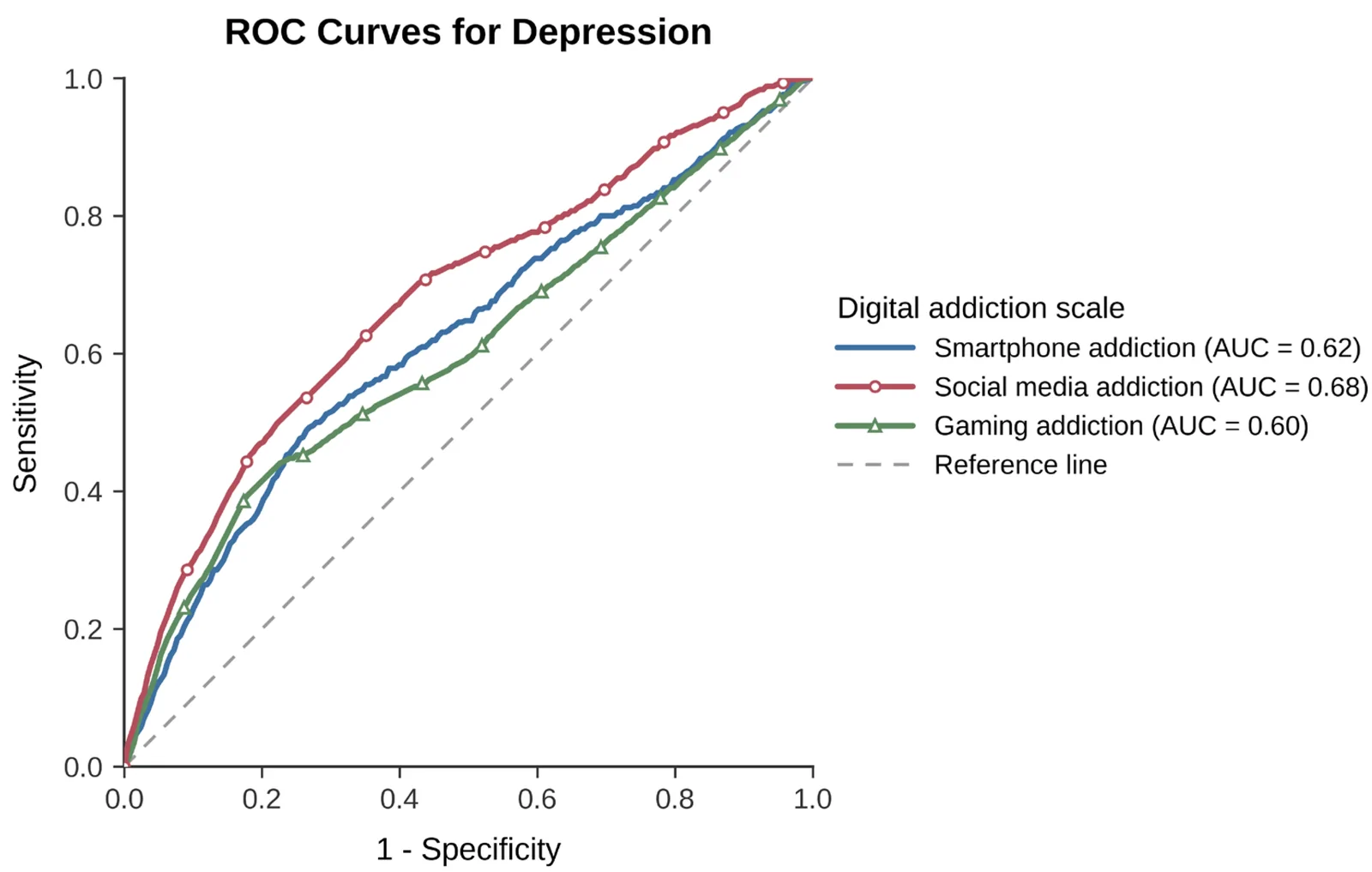
ROC curves of digital technology addiction for depression.

For depression, BSMAS had the highest AUC, although discrimination remained poor (AUC = 0.68, 95% CI 0.63–0.73; *p* < 0.001). SABAS (AUC = 0.62, 95% CI 0.57–0.66; *p* < 0.001) and GAS (AUC = 0.60, 95% CI 0.55–0.65; *p* < 0.001) also showed poor discrimination (Figure 2).

**Figure 3:**
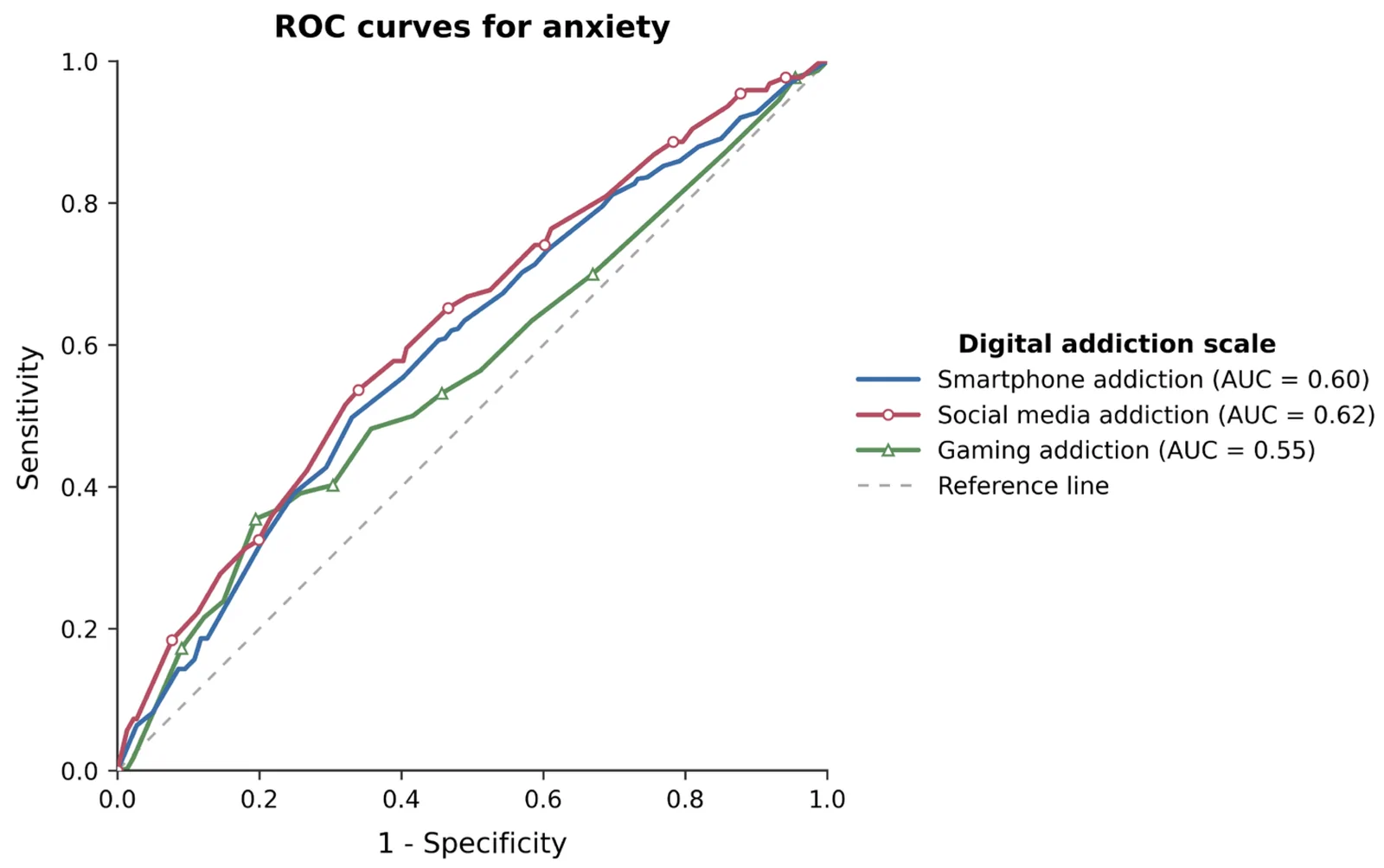
ROC curves of digital technology addiction for anxiety.

For anxiety, BSMAS (AUC = 0.62, 95% CI 0.57–0.67; *p* < 0.001) and SABAS (AUC = 0.60, 95% CI 0.55–0.64; *p* < 0.001) showed poor discrimination. GAS was close to chance performance (AUC = 0.55, 95% CI 0.51–0.60; *p* < 0.05), indicating negligible practical value despite nominal statistical significance (Figure 3).

For stress, the SABAS threshold of ≥23.5 had a sensitivity of 51.0% and specificity of 85.4%. The BSMAS threshold of ≥17.5 increased sensitivity to 59.0% but reduced specificity to 67.7%. The GAS threshold of ≥15.5 had a sensitivity of 42.0% and specificity of 82.1%.

For depression, sensitivity ranged from 41.0% to 50.0% and specificity from 73.2% to 80.1%. The BSMAS threshold of ≥18.5 provided the highest AUC, with sensitivity of 50.0% and specificity of 76.9%.

For anxiety, sensitivity ranged from 35.0% to 52.1% and specificity from 67.2% to 80.9%. The GAS threshold of ≥15.5 had the lowest sensitivity (35.0%) and an AUC close to chance. Full operating characteristics are presented in Table 3.

## 4. Discussion

### 4.1. Principal Findings and Conceptual Interpretation

This study assessed whether three digital-use scales could distinguish normal from non-normal DASS-21 symptom classifications among medical students. SABAS showed fair discrimination for stress (AUC = 0.75), whereas all other AUCs were poor or close to chance. The candidate thresholds generally had higher specificity than sensitivity, but sensitivity remained low (35.0–59.0%), indicating that many students with non-normal DASS-21 scores would be missed. These findings support, at most, an adjunctive role for digital-use scores and do not support their use as independent mental health screening or diagnostic instruments. The conceptual distinctions between broad smartphone dysregulation, platform-specific social media use, and gaming-related problems may partly explain their differing relationships with distress [11,12,13,14,15].

### 4.2. Prevalence and Distribution of Distress Symptoms

A substantial minority of participants had non-normal DASS-21 scores, but most non-normal classifications were mild. This distribution is consistent with evidence that psychological symptoms are frequent among medical students, while the severity and clinical significance vary across populations and measurement approaches [7,8,9,10,33]. The predominance of mild symptoms is important because DASS-21 categories indicate symptom severity rather than a psychiatric diagnosis.

Vietnamese evidence also indicates that psychological distress occurs in health-related occupational groups, although prevalence estimates are not directly comparable because populations and instruments differ [34]. The present results, therefore, reinforce the need for accessible student support pathways while also emphasizing that symptom questionnaires and behavioral indicators cannot replace clinical assessment.

### 4.3. Interpretation of Discriminative Performance and External Comparisons

The strongest result was the fair discrimination of SABAS for stress. This finding is compatible with the literature linking problematic smartphone use with stress, depression, anxiety, and sleep disturbance [1,2,4]. By contrast, BSMAS and GAS showed poor discrimination across outcomes, despite established associations between social media or gaming behaviors and psychological well-being [3,35,36,37]. Association and discrimination are different properties: a behavior may correlate with distress, yet still perform poorly when used to classify individual students.

The observed AUCs of 0.55–0.75 should also be interpreted in relation to the reference standard. Clinical validation studies compare an index measure with an independently administered diagnostic assessment; for example, the Vietnamese DASS-21 validation used psychiatrist-administered Structured Clinical Interviews [20]. In the present study, both the digital-use scales and DASS-21 were self-reported and measured different constructs. The analyses, therefore, estimated concordance with symptom categories rather than diagnostic accuracy, making modest AUCs methodologically unsurprising and limiting direct comparison with clinically anchored validation studies.

The GAS anxiety result requires particular caution. An AUC of 0.55 indicates near-chance discrimination, and its nominal *p*-value should not be interpreted as evidence of practical usefulness. Gaming may represent problematic engagement for some students but recreation or coping for others [17,35,36]. Restricted variability may also have contributed because severe anxiety was uncommon. These explanations remain tentative and require confirmation in longitudinal studies.

### 4.4. Theoretical Integration: Compensatory Internet Use

Compensatory Internet Use Theory provides one possible framework for interpreting the observed co-variation [17]. Students experiencing negative affect may turn to smartphones, social media, or games as coping strategies, and repeated use may subsequently become dysregulated. The reverse pathway is also plausible, and shared factors such as sleep disruption, academic pressure, or social isolation may influence both digital behavior and distress. Because the study was cross-sectional, temporal direction and causality cannot be established.

### 4.5. Public Health and Institutional Implications

The higher specificity than sensitivity at most thresholds does not mean that the scales can rule out psychological distress. On the contrary, the low sensitivity indicates that a substantial proportion of students with non-normal DASS-21 scores would be missed. The thresholds are therefore unsuitable as stand-alone first-stage screening tools. At most, a high digital-use score could serve as a supplementary behavioral flag prompting assessment with a validated mental health questionnaire or, where indicated, clinical evaluation. PPV and NPV are population-dependent, and institutional implementation should not be considered until the thresholds have been externally validated.

### 4.6. Strengths and Limitations

This study compared three forms of problematic digital engagement within the same medical-student sample and explicitly distinguished symptom-based discrimination from clinical diagnostic accuracy. Several limitations must nevertheless be considered. First, the cross-sectional design precluded causal or temporal inference. Second, the single-center setting limited generalisability. Third, the analytical sample represented complete responders rather than the full randomly invited sample, creating potential self-selection and attrition bias. Fourth, household economic status was recorded only descriptively and was not included as a covariate; other potential confounders were also not evaluated in the ROC analyses. Fifth, formal adjustment for multiple comparisons was not performed, so borderline *p*-values require caution. Sixth, SABAS and the six-item GAS adaptation did not have direct, formally established Vietnamese validation evidence, although internal consistency was assessed. Seventh, DASS-21 was a self-report symptom measure rather than an independent clinical reference standard. 

Finally, thresholds were derived and evaluated in the same dataset, which may overestimate apparent performance. External or split-sample validation is required before these candidate cut-offs can be considered for practice. Future validation studies should use an independent clinical reference standard and follow the STARD 2015 reporting recommendations [38].

## 5. Conclusions

Among the medical students surveyed, non-normal DASS-21 symptom scores were common but predominantly mild. The digital-use scales showed modest discrimination, with fair performance only for SABAS in relation to stress and near-chance performance for GAS in relation to anxiety. Because sensitivity was low and the reference classification was self-reported, the ROC-derived thresholds should be regarded as preliminary behavioral proxies rather than stand-alone screening or diagnostic criteria. Independent multicenter validation, preferably using longitudinal designs and clinically anchored outcomes, is required before any institutional application.

## Figures and Tables

**Table 1:** Socio-demographic characteristics of the study participants (*n* = 600).

Variable	Category/Statistic	*n*	%
Age (years)	Mean ± SD (range)	22.9 ± 3.7 (20–48)	
Gender	Male	284	47.3
	Female	316	52.7
School year	Second year	69	11.5
	Third year	247	41.2
	Fourth year	176	29.3
	Fifth year	108	18.0
Economic status	Low income	21	3.5
	Adequate income	352	58.7
	High income	227	37.8

SD: standard deviation.

**Table 2:** Prevalence and severity levels of stress, depression, and anxiety symptoms (*n* = 600).

Outcome	Severity	*n*	%
Stress	Normal	418	69.6
	Mild	106	17.7
	Moderate	64	10.7
	Severe	8	1.3
	Extremely severe	4	0.7
Depression	Normal	373	62.2
	Mild	144	24.0
	Moderate	69	11.5
	Severe	8	1.3
	Extremely severe	6	1.0
Anxiety	Normal	366	61.0
	Mild	159	26.5
	Moderate	62	10.3
	Severe	10	1.7
	Extremely severe	3	0.5

**Table 3:** ROC-derived cut-off values of digital technology addiction metrics for psychological distress domains.

Outcome	Index Scale	AUC (95% CI)	Cut-Off	Sen, % (95% CI)	Spec, % (95% CI)	PPV (%)	NPV (%)
Stress	SABAS	0.75 (0.70–0.80)	≥23.5	51.0 (43.9–58.3)	85.4 (81.7–88.5)	60.4	80.0
	BSMAS	0.67 (0.62–0.71)	≥17.5	59.0 (51.5–65.7)	67.7 (63.1–72.0)	44.2	79.1
	GAS	0.64 (0.60–0.70)	≥15.5	42.0 (35.4–49.6)	82.1 (78.1–85.4)	50.7	76.6
Depression	SABAS	0.62 (0.57–0.66)	≥21.5	49.0 (42.5–55.4)	73.2 (68.5–77.4)	52.6	70.2
	BSMAS	0.68 (0.63–0.73)	≥18.5	50.0 (43.8–56.7)	76.9 (72.4–80.9)	57.0	71.8
	GAS	0.60 (0.55–0.65)	≥14.5	41.0 (34.8–47.5)	80.1 (75.8–83.9)	55.7	69.1
Anxiety	SABAS	0.60 (0.55–0.64)	≥20.5	49.1 (42.8–55.5)	67.5 (62.5–72.1)	49.1	67.5
	BSMAS	0.62 (0.57–0.67)	≥17.5	52.1 (45.8–58.5)	67.2 (62.2–71.8)	50.4	68.7
	GAS	0.55 (0.51–0.60)	≥15.5	35.0 (29.2–41.4)	80.9 (76.5–84.6)	53.9	66.1

AUC: area under the curve; CI: confidence interval; Sen: sensitivity; Spec: specificity; PPV: positive predictive value; NPV: negative predictive value.

## Data Availability

The data supporting the findings of this study are available from the corresponding author, L.M.D., upon reasonable request.

## Abbreviations

The following abbreviations are used in this manuscript:

AUC	Area under the curve
CI	Confidence intervals
DASS-21	Depression Anxiety Stress Scales-21
NPV	Negative predictive value
PPV	Positive predictive value
ROC	Receiver operating characteristic
SD	Standard deviation
